# Comparative analysis of Rb1, P16 and ER as diagnostic, prognostic and potential targets for therapeutic agents in ovarian epithelial tumors: an immunohistochemical study of 130 ovarian carcinomas

**DOI:** 10.1186/s13048-015-0163-1

**Published:** 2015-06-05

**Authors:** Donna Catherine Ferguson, Daniel Jerad Long, Megan Christine Smith, Laura Deeanne Craig-Owens, Julie Means, Oluwole Fadare, Mohamed Mokhtar Desouki

**Affiliations:** Department of Pathology Microbiology and Immunology, Vanderbilt University School of Medicine, 1161 21st Avenue South, MCN, C-2310A, Nashville, TN 37232-2561 USA; Department of Tennessee Oncology, Sarah Cannon Research Institute, Franklin, TN USA; Department of Pathology, University of California San Diego, San Diego, CA USA

**Keywords:** Rb1 protein, P16, ER, Ovarian carcinoma, Immunohistochemistry

## Abstract

**Background:**

Deregulation of CDK4/6, cyclin D/P16 and retinoblastoma (Rb) are known aberrations in certain malignancies. There has been a recent interest in exploring the combination of letrozole and CDK4/6 inhibitors in recurrent ER+ ovarian cancers.

**Methods:**

This study aimed to determine the frequency of expression of Rb1, P16 and ER in ovarian epithelial tumors by immunohistochemistry.

**Results:**

Co-expression of all 3 markers studied was seen in 10 % of high grade serous carcinoma (HGSC) and low grade serous carcinoma (LGSC). Coordinate expression of Rb1+ and ER+ in HGSC and LGSC was seen in 67 % of grade 1/2 vs. 44 % of grade three tumors (p < 0.05). The reverse was true with positive P16 staining in 73 % of grade three vs. 32 % of grade 1/2 tumors (p < 0.001).

**Conclusions:**

Coordinate pattern of Rb1+ and ER+ in HGSC and LGSC is 19 and 50 %, respectively. Rb1 and P16 show inverse expression pattern according to tumor grade with more frequent Rb1 in low grade vs. more frequent P16 in grade 3 tumors. These data provide a rational basis for clinical trials that aim to target these proteins.

## Background

Ovarian cancer is the eighth most common cancer, the fifth leading cause of cancer deaths in women and the number one leading cause of cancer related deaths of the female reproductive system. In 2014, 21,980 women in the United States were diagnosed with ovarian cancer and 14,270 women died from ovarian cancer [1]. Overall, ~ 80 % of patients diagnosed with ovarian epithelial cancer will initially respond then relapse after first-line platinum and taxane-based chemotherapy and may benefit from subsequent therapies [2, 3]. When ovarian cancer is found in its early stages, treatment is most effective.

Median survival for patients with recurrent ovarian cancer is 25–27 months. Clinical recurrences that take place within 6 months of completion of a platinum-containing regimen are considered platinum-resistant. Alternative treatments for these patients include anthracyclines, taxanes, topotecan and gemcitabine. Patients with platinum-resistant disease who fail 1–2 lines of therapy should be encouraged to be enrolled in clinical trial [2].

Advances in the understanding of the molecular pathogenesis of ovarian cancer coupled with the development of novel-targeted therapies are needed to improve outcomes. Deregulation of the CDK4/6–cyclin D/P16– retinoblastoma (Rb) signaling pathway is among the most common aberrations found in human cancer. In the case of ovarian cancer, P16 expression is most commonly altered due to promoter methylation, and less commonly by homozygous deletion or mutation [4].

PD-0332991 is a selective inhibitor of the CDK4/6 kinases with the ability to block Rb phosphorylation [5]. Concentration-dependent antiproliferative effects of PD-0332991 were seen in all ovarian cancer cell lines, but varied significantly between individual lines. Rb-proficient cell lines with low P16 expression were most responsive to CDK4/6 inhibition. Copy number variations of *CDKN2A*, *RB*, *CCNE1*, and *CCND1* were associated with response to PD-0332991. Rb-proficiency with low P16 expression was seen in 97/262 (37 %) of ovarian cancer patients and was independently associated with poor progression-free survival (PFS) [4].

Synergism with antiestrogen therapy and CDK4/6 inhibition has recently been demonstrated beneficial advantage in advanced estrogen receptor positive (ER+) breast cancer. For women with ER+ stage IV breast cancer treated with the combination of palbociclib (CDK4/6 inhibitor) plus letrozole, the median PFS was 20.2 months, a statistically significant improvement compared to the 10.2 months of PFS in women who received letrozole alone (HR = 0.488 [95 % CI: 0.32, 0.75]; p < 0.001 [6].

In a large-scale study, 36 % of ovarian cancers were ER+. Estrogen stimulates tumor growth *via* ER. Antiestrogens, such as tamoxifen, block the ER pathway, and aromatase inhibitors such as letrozole directly inhibit the synthesis of estrogen. In theory, both antiestrogens and aromatase inhibitors should exhibit antitumor effects against ovarian cancer [7].

In a study by Smyth *et al.*, 42 ER+ recurrent ovarian cancer patients received letrozole 2.5 mg/day orally. Of the 33 patients who had a measurable lesion, three patients (9 %) achieved partial remission and 14 patients (42 %) maintained stable disease state for 12 weeks. The study showed a positive correlation between the level of ER expression and treatment response [8].

There has been a recent interest to explore the combination of letrozole plus CDK 4/6 inhibitor in recurrent ER+ ovarian epithelial cancer and a proposed phase I/II clinical trial is being written. The specific aim of this study is to determine the frequency of expression of ER, Rb1 and P16 by immunohistochemistry (IHC) in tissue sections prepared from formalin fixed, paraffin embedded tissue blocks of ovarian epithelial tumors. These data provide a rational basis for clinical trials that aim to target these proteins.

## Results

**Fig. 1:**
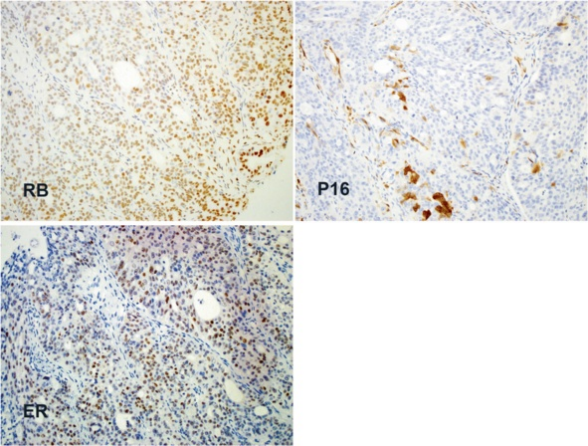
Representative case of ovarian high grade serous carcinoma stained with Rb1, P16 and ER by immunohistochemistry. Ovarian epithelial carcinomas of different histologic subtypes and grades in tissue microarray slide were used. Anti-Rb1 rabbit polyclonal, anti-P16 and mouse anti-human ER primary antibodies were used. Note strong positive expression of Rb1, negative P16 and positive ER (H-score of 80)

Table 1 summarizes the frequency of individual markers and coordinate patterns of expression in ovarian epithelial tumors studied. Co-expression of all three markers (Rb1, P16 and ER) was seen in 10, 10, 6 and 0 % of high grade serous carcinoma (HGSC), low grade serous carcinoma (LGSC), endometrioid carcinoma (EC) and mucinous carcinomas, respectively. In contrast, coordinate negative expression of all three markers was seen in 32 % of mucinous carcinomas compared to 4, 10 and 6 % in HGSC, LGSC and ECs, respectively (p < 0.05). In HGSC, the expression pattern of positive P16 was seen in 67 % of cases compared to 30, 62 and 11 % in LGSC, EC and mucinous carcinomas, respectively (p < 0.05) (Fig. 1).

**Table 1:** Single and coordinate pattern of Rb1, P16 and ER expression in ovarian epithelial carcinoma cases studied (n = 130)

Tumor type	Rb1+	P16+	ER-	Rb1+/ P16+	Rb1+/ P16-	Rb1+/ ER+	P16+/ ER+	Rb1+/ P16+/ ER+	Rb1-/ P16-/ ER-
High grade serous [HGSC] (n = 67)	36 (54 %)	45 (67 %)	30 (45 %)	20 (30 %)	16 (24 %)	13 (19 %)	21 (31 %)	7 (10 %)	3 (4 %)
Low grade serous [LGSC] (n = 10)	7 (70 %)	3 (30 %)	7 (70 %)	2 (20 %)	5 (50 %)	5 (50 %)	2 (20 %)	1 (10 %)	1 (10 %)
Endometrioid (EC) (n = 34)	16 (47 %)	21 (62 %)	17 (50 %)	7 (21 %)	9 (26 %)	2 (6 %)	12 (35 %)	2 (6 %)	2 (6 %)
Mucinous carcinoma (MC) (n = 19)	11 (58 %)	2 (11 %)	3 (16 %)	1 (5 %)	10 (52 %)	1 (5 %)	1 (5 %)	0	6 (32 %)
Total (n = 130)	70 (54 %)	71 (55 %)	57 (44 %)	30 (23 %)	40 (31 %)	17 (13 %)	36 (28 %)	10 (8 %)	12 (9 %)

**Fig. 2:**
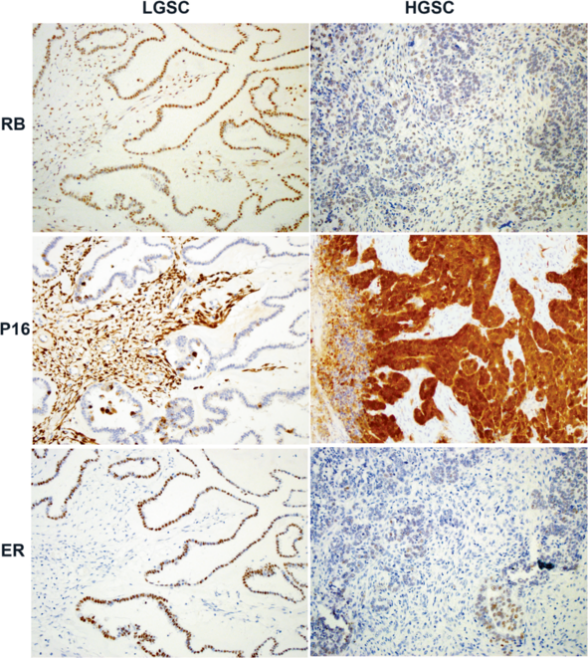
Rb1, P16 and ER expression according to tumor grade in representative cases of ovarian serous carcinomas. Ovarian epithelial carcinomas of different histologic subtypes and grades in tissue microarray slide were used. Anti-Rb1 rabbit polyclonal, anti-P16 and mouse anti-human ER primary antibodies were used for immunohistochemistry. Notice high nuclear expression of Rb1 in low grade serous carcinoma (LGSC) compared to weak positive staining in high grade serous carcinoma (HGSC). The reverse is true for p16 with high expression in HGSC compared to negative staining in LGSC. ER shows high expression score in LGSC compared to low score in HGSC

The co-expression of Rb1+ and ER+ was seen in 19 % of HGSC cases compared to 10, 6 and 5 % in LGSC, EC and mucinous carcinomas, respectively (p < 0.05). Negative P16 stain was seen in 89 and 70 % mucinous carcinomas and LGSC, respectively with statistically significant difference (p < 0.05) in comparison to 33 % negative HGSC and 38 % negative ECs. ER positive stain was observed in 70, 50, 45 and 16 % of LGSC, EC, HGSC, and mucinous carcinomas, respectively (Fig. 2).

There was no significant difference in the distribution of any of the markers with tumor size (pT) and lymph node status (pN). Rb1 was positive in 38/57 (67 %) of grade 1/2 vs. 32/73 (44 %) of grade three tumors (p < 0.05). P16 was positive in 53/73 (73 %) of grade three vs. 18/57 (32 %) of grade 1/2 tumors (p < 0.001) (Fig. 2) (Table 2).

**Table 2:** Rb1, P16 and ER protein expression in ovarian epithelial carcinoma cases studied according to clinico-pathologic variables (n = 130)

IHC expression	Tumor size (pT)			Tumor grade			
	T1 (n = 51)	T2 (n = 53)	T3 (n = 19)	G1 (n = 28)	G2 (n = 29)	G3 (n = 73)	G1/2 (n = 57)
Rb1+ (n = 70)	24	33	9	19	19	32	38
P16+ (n = 71)	27	27	13	7	11	53	18
ER+ (n = 57)	23	24	9	10	9	38	19
Rb1+/P16+ (n = 30)	9	11	8	3	5	19	8
Rb1+/P16- (n = 40)	18	24	1	10	16	14	26
Rb1-/P16+ (n = 41)	19	16	5	2	6	33	8
Rb1+/ER- (n = 46)	14	23	6	12	15	19	27
Rb1+/ER+ (n = 24)	10	10	3	7	4	13	11
Rb1+/P16+/ER+ (n = 10)	4	2	3	2	1	7	3
Rb1-/P16-/ER- (n = 12)	8	2	1	6	2	4	8

There is a positive correlation between P16 and ER expression (r = 0.3), P16 and tumor size (r = 0.2) and P16 and tumor grades (r = 0.4). There is a negative correlation between Rb1 and P16 (r = −0.12) and Rb1 and tumor grades (r = −0.2).

## Discussion

We studied Rb1, P16 and ER protein expression in a relatively large cohort of ovarian epithelial tumors of varying histotypes and grades to evaluate the frequency and patterns of expression and correlate with clinico-pathologic parameters. Rb1 functional pathway abnormalities have been reported to lead to P16 over-expression in dysplastic and neoplastic tissue e.g. in tumors of the lower genital tract due to high-risk types of human papillomavirus which lead to inactivation of Rb1 [9]. As reported by others, a negative correlation between Rb1 and P16 expression in our cohort has been identified [9, 10].

High expression of P16 could be explained by abrogation of functional Rb1 signaling. A cell with compromised Rb1 pathway will induce an over-expression of P16 due to abnormalities in the negative feedback of the Rb1 especially in high grade tumors with aggressive behavior [11]. On the other hand, negative expression of P16 is common in low grade and less aggressive tumors such as mucinous carcinomas or ECs [4]. In contrast to strong p53 protein expression in most cases with mutated p53 gene, cases with complete absence of Rb1 staining may be an indicator of Rb1 function silencing [12]. The significance of increased expression of Rb1 protein even in the presence of intact tumor suppressor protein is not known [12–14].

The coordinate pattern of negative stain for the three markers utilized in the current study namely Rb1, P16 and ER was seen in 32 % of mucinous carcinomas in contrast to 4, 10, and 10 % of HGSC, LGSC, and ECs, respectively. Konecny *et al.*, reported 37 % of primary ovarian cancer patients demonstrated Rb1 proficiency with low P16 expression with poor clinical outcome but were most likely to benefit from CDK4/6 inhibition [4].

Co-expression of Rb1+ and ER+ has been identified in 19 and 50 % of HGSC and LGSC, respectively with statistically significant difference (p < 0.05). The intensity of ER (H-score) is high in LGSC and low in HGSC with statistically significant difference (Table 3). This group of patients (Rb1+, ER+) could be a target for CDK4/6 inhibition plus letrozole, with or without antiestrogen therapy, a regimen with proven efficacy in patients with advanced ER+ breast cancer [15, 16]. The final results of a randomized phase two study reported a statistically significant improvement of the median progression free survival (PFS) of 20.2 months for women with ER+ stage IV breast cancer treated with combination of palbociclib (CDK4/6 inhibitor) plus letrozole compared to 10.2 months of PFS for women who received letrozole alone [6].

**Table 3:** Rb1 expression according to ER H-score by IHC in high and low grade ovarian serous carcinomas

Tumor type	HGSC n = 67 (p-value*)	LGSC n = 10 (p-value*)
Rb1+/ER- (H-score 0–25)	23/67 (34 %) (P = 0.16)	2/10 (20 %) (p = 0.26)
Rb1+/ER (H-score 26–75)	4/67 (6 %) (p = 0.02)	0 (p = 1)
Rb1+/ ER (H-score 76–150)	8/67 (12 %) (p = 0.03)	1/10 (10 %) (p = 1)
Rb1+/ ER (H-score > 150)	2/67 (3 %) (p = 0.61)	3/10 (30 %) (<0.001)

Abbreviations: *HGSC* High grade serous carcinoma, *LGSC* Low grade serous carcinoma. *Chi square or Fisher exact test comparing HGSC or LGSC against all ovarian epithelial carcinomas studied (n = 130)

No statistically significant difference has been identified in the expression of the markers studied either individually or coordinate patterns according to tumor size (pT) and lymph node status (pN). However, similar to other studies, our findings indicated that there is an inverse correlation between the Rb1 and P16 expression according to tumor grade with high expression of the Rb1 in low grade tumors in contrast to high expression of P16 in high grade lesions (Fig. 2) [4, 9].

In our study, 10/130 (8 %) showed complete absence of Rb1 staining. The cases were HGSC (5/67; 7 %), EC (3/34; 9 %), mucinous carcinomas (2/19; 11 %). Armes *et al.* reported similar findings with complete absence of Rb1 in 9 % of HGSC and others reported persistent expression of Rb1 in most cases even with hemizygous deletions at the Rb1 locus in ovarian cancer [9, 17].

In conclusion, coordinate pattern of Rb1+ and ER+ in HGSC and LGSC is 19 and 50 %, respectively. Rb1 and P16 show inverse expression pattern according to tumor grade with more frequent Rb1 in low grade vs. more frequent P16 in grade three tumors. These data provide a rational basis for clinical trials that aim to target these proteins.

## Methods

### Tissue microarray (TMA)

This study was approved by Vanderbilt University School of Medicine institutional review board. Ovarian epithelial carcinomas of different histologic subtypes and grades (n = 130) as well as normal tissue as a control (n = 8) in TMA slides were used. The TMA contained 68 HGSC, 10 LGSC, 34 EC and 19 mucinous carcinomas.

### Immunohistochemistry

Rb1, P16 and ER expression were determined by IHC. TMA slides were stained on the Leica Bondmax platform (Leica Microsystems, Buffalo Grove, IL). Antigen retrieval was performed on the instrument utilizing Epitope Retrieval Solution 2 (EDTA based proprietary reagent, Leica Microsystems Cat# AR9640) for 20 min. Anti-Rb1 rabbit polyclonal (LSBio Cat#LS-B1495, 1:200 dilution), anti-P16 ready-to-use (CINTECH/Roche) and mouse anti-human ER ready-to-use (Clone 6 F11, Leica Microsystems, Buffalo Grove, IL) primary antibodies were used. Anti-Rb1 antibody was applied for 60 min, followed by an anti-rabbit polymer. Anti-P16 was applied for 60 min, followed by a mouse anti-rabbit secondary antibody and then a tertiary anti-rabbit polymer. Anti-ER was applied for 15 min, followed by a rabbit anti-mouse secondary antibody and then a tertiary anti-rabbit polymer. Endogenous peroxidases were blocked using 3 % hydrogen peroxide. TMA slides were then stained with 3, 3’-diaminobenzidine tetrahydrochloride (DAB) chromogen and counterstained in hematoxylin for visualization.

Only nuclear staining for Rb1 was considered to be positive and scored as: negative; ≤5 % staining, weak staining (1+); weak intensity in >6 % and/or focal strong intensity (≤25 %) simulating expression in normal control tissue, and strong positive (2+); diffuse strong intensity (>25 %) [4]. For the purpose of analysis in this study, only Rb1 with strong (2+) intensity is considered positive, unless stated otherwise. Strong and diffuse nuclear and/or cytoplasmic staining was considered positive for P16 expression. Quantification of nuclear staining using the H-scoring system was used to evaluate the ER expression [18] with cases scored ≤25 as one group (negative for the purpose of analysis) and cases scored >25 as another group (positive). ER+ cases were subdivided into mild (H-Score 26–75), moderate (H-score 76–150) and strong positive (H-score >150).

### Statistical analysis

A linear correlation, the Chi-square and Fisher’s exact tests to determine correlation and significant difference between different variables were performed. The IHC scores were considered nominal to calculate significance. P < 0.05 was considered significant.

## Abbreviations

EC: Endometrioid carcinoma
HGSC: High grade serous carcinoma
IHC: Immunohistochemistry
LGSC: Low grade serous carcinoma
Rb1: Retinoblastoma −1
TMA: Tissue microarray
